# Can Population Modelling Principles be Used to Identify Key PBPK Parameters for Paediatric Clearance Predictions? An Innovative Application of Optimal Design Theory

**DOI:** 10.1007/s11095-018-2487-1

**Published:** 2018-09-14

**Authors:** Elisa A. M. Calvier, Thu Thuy Nguyen, Trevor N. Johnson, Amin Rostami-Hodjegan, Dick Tibboel, Elke H. J. Krekels, Catherijne A. J. Knibbe

**Affiliations:** 1 Division of Systems Biomedicine and Pharmacology, Leiden Academic Centre for Drug Research (LACDR), Gorlaeus Laboratories, Leiden University, Einstein weg 55, 2333 CC Leiden, The Netherlands; 2 IAME, UMR 1137, INSERM, University Paris Diderot, Sorbonne Paris Cité, Paris, France; 3 Simcyp Limited, Sheffield, UK; 4 Manchester Pharmacy School, University of Manchester, Manchester, UK; 5 Intensive Care and Department of Pediatric Surgery, Erasmus University Medical Center - Sophia Children’s Hospital, Rotterdam, The Netherlands; 6 Department of Clinical Pharmacy, St. Antonius Hospital, Nieuwegein, The Netherlands

**Keywords:** paediatrics, physiologically-based pharmacokinetic, population modelling, population optimal design

## Abstract

**Purpose:**

Physiologically-based pharmacokinetic (PBPK) models are essential in drug development, but require parameters that are not always obtainable. We developed a methodology to investigate the feasibility and requirements for precise and accurate estimation of PBPK parameters using population modelling of clinical data and illustrate this for two key PBPK parameters for hepatic metabolic clearance, namely whole liver unbound intrinsic clearance (CLint_,u,WL_) and hepatic blood flow (Qh) in children.

**Methods:**

First, structural identifiability was enabled through re-parametrization and the definition of essential trial design components. Subsequently, requirements for the trial components to yield precise estimation of the PBPK parameters and their inter-individual variability were established using a novel application of population optimal design theory. Finally, the performance of the proposed trial design was assessed using stochastic simulation and estimation.

**Results:**

Precise estimation of CLint_,u,WL_ and Qh and their inter-individual variability was found to require a trial with two drugs, of which one has an extraction ratio (ER) ≤ 0.27 and the other has an ER ≥ 0.93. The proposed clinical trial design was found to lead to precise and accurate parameter estimates and was robust to parameter uncertainty.

**Conclusion:**

The proposed framework can be applied to other PBPK parameters and facilitate the development of PBPK models.

**Electronic supplementary material:**

The online version of this article (10.1007/s11095-018-2487-1) contains supplementary material, which is available to authorized users.

## Introduction

Physiologically-based pharmacokinetic (PBPK) models are an essential tool to predict the pharmacokinetics (PK) of new compounds in various human populations. PBPK models quantify how drug molecules, characterized by drug-specific parameters reflecting their properties, interact with organisms which are defined by system-specific parameters reflecting anatomical and physiological measures. Predictions are made by feeding drug-specific parameters into a PBPK model with the system-specific parameter values of the population of interest. This has been proven useful for instance to define first-in-child doses (1) (2) (3) or support paediatric clinical trial design. (2) (4).

System-specific parameter values for PBPK models can be either obtained experimentally by direct measurements or they can be derived from clinical PK data through model parameter estimation (5–7). The latter not only allows for coping with a lack of experimental data, which may be particularly relevant in special patient populations, but is also the most robust approach which has been well described by Tsamandoura *et al*. (8). For instance, it has been found that ontogeny functions estimated from clinical PK data performed markedly better than those developed from *in vitro* measurements (7). However, estimating parameter values is not always trivial due to structural identifiability issues. Structural identifiability refers to the possibility to uniquely estimate model parameters given a model and ideal, error-free data (9,10). Without a guarantee of structural identifiability, parameter estimates will be either non obtainable or random and unreliable (11). In such cases global structural identifiability, meaning that only one unique solution exists for each parameter, can be obtained by fixing or using priors for some model parameters, while estimating the others.

Once global structural identifiability of model parameters is achieved, population modelling of longitudinal data can be used for estimation of the PBPK parameters and their inter-individual variability. However, structural identifiability of a model does not guarantee precise and unbiased parameter estimates, as this depends on the information content of the data. Therefore, model parameters should also be numerically identifiable, meaning that accurate and precise estimates can be obtained given real, observed data. Numerical identifiability can in general terms be achieved by designing studies that yield sufficiently rich data (12). Evaluation and optimization of trial designs for population PK analyses can be achieved without time-consuming clinical trial simulations using optimal design software (13). Optimal design software packages approximate the Fisher information matrix (FIM) of population models and rely on the Rao and Cramer bound that states that the inverse of the FIM is the lower bound of the variance-covariance matrix of any unbiased estimator of the parameters. While to date population optimal design is routinely used in optimizing trials for the precise estimation of population PK parameters (14), it has never been applied to the estimation of population PBPK parameters.

**Fig. 1:**
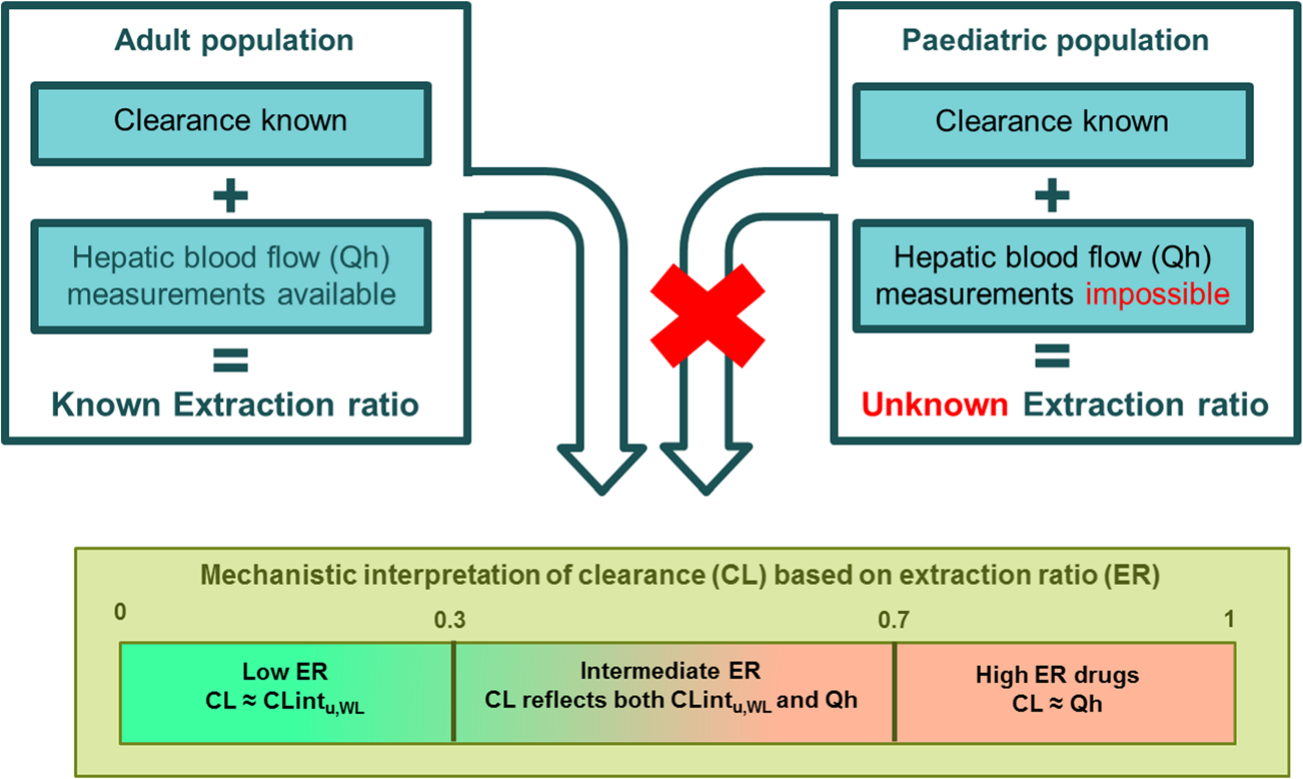
The relationships between extraction ratio (ER), whole liver unbound intrinsic clearance (CLint_,u,WL_) and hepatic blood flow (Qh) for the adult and paediatric population. Without knowing Qh, the ER of a drug is unknown, making it mathematically impossible to disentangle Qh and CLint_,u,WL_ from clinical concentration-time data of one drug. CL: is the blood clearance of the unbound drug, see equations 1–6.

For drugs undergoing hepatic metabolism, the part of a paediatric PBPK model describing this clearance contains two key parameters that cannot be directly measured and that cannot be simultaneously estimated from the PK data of one drug, due to identifiability issues, namely whole liver unbound intrinsic clearance (CLint_,u,WL_) and hepatic blood flow (Qh). As illustrated in Fig. 1, both contribute to the overall hepatic metabolic clearance. The extraction ratio (ER) of a drug reflects the relative contribution of these two parameters to hepatic metabolic clearance. On one hand, clearance of high ER drugs reflects Qh, while clearance of low ER drugs reflects CLint_,u,WL_. To achieve global structural identifiability, Qh is often fixed while CLint_,u,WL_ is estimated based on PK data (5,15). As Qh cannot be directly measured in very young children, its value is often fixed to an assumed percentage of cardiac output (5,15). When this assumed percentage is however not correct, the estimation of CLint_,u,WL_ values and enzyme ontogeny functions may be biased. Therefore, there is a need for other methodologies that allow us to cope with the lack of experimental data on system-specific parameters. Even though paediatric PBPK models have been proven to predict clearance of many drugs with reasonable accuracy (15–17), such methods would ultimately improve and accelerate the development and validation of these models in various paediatric populations.

The aim of this paper was to develop an analysis framework to investigate whether population modelling approach can be used to estimate PBPK model parameters from clinical PK data and establish the required criteria for such estimations. The developed analysis framework depends on firstly establishing the data requirements for structural identifiability of PBPK model parameters. Then on a subsequent application of innovative population optimal design theory to define a clinical trial design that yields precise estimates of the relevant model parameters. And lastly on the evaluation of the performance of the proposed trial design in terms of bias and imprecision of parameter estimates using stochastic simulation and estimation. The approach is illustrated using the simultaneous estimation of Qh and CLint_,u,WL_ in the paediatric population as an example.

## Materials and Methods

An analytical workflow was developed, which is composed of the three following steps: structural identifiability, optimal design and evaluation of the optimized design performance. R version 3.3.1 was used for calculations, data management and visualizations (18). For optimal design procedures of step 2 of the analytical workflow, the PFIM program 4.0 running in R was used (19). PBPK simulations were performed with Simcyp software (Simcyp, Sheffield, UK) V15.R1 to derive PBPK model parameters value for optimal design procedures in step 2 and the stochastic simulations and estimations in step 3. Stochastic simulations and estimations were performed in step 3 using NONMEM version 7.3 (20) and Perl-speaks-NONMEM software package version 4.6.0 (21).

### Step 1: Structural Identifiability

The dispersion model (equations 1–6) was used to describe hepatic plasma clearance based on PBPK-principles. This model was selected as it has been reported to better predict clearance than the well-stirred model for high clearance drugs, while both models lead to equivalent clearance predictions for other drugs (22,23).

1 $$ \mathrm{CLp}=\mathrm{CLb}\times \mathrm{B}:\mathrm{P} $$

2 $$ \mathrm{CLb}=\mathrm{Qh}\times \mathrm{ER} $$

3 $$ \mathrm{ER}=1-{\mathrm{F}}_{\mathrm{H}} $$

4 $$ {\mathrm{F}}_{\mathrm{H}}=\frac{4\mathrm{a}}{{\left(1+\mathrm{a}\right)}^2\exp \left\{\left(\mathrm{a}-1\right)/2{\mathrm{D}}_{\mathrm{N}}\right\}-{\left(1-\mathrm{a}\right)}^2\exp \left\{-\left(\mathrm{a}+1\right)/2{\mathrm{D}}_{\mathrm{N}}\right\}} $$

5 $$ \mathrm{a}={\left(1+4{\mathrm{R}}_{\mathrm{N}}\times {\mathrm{D}}_{\mathrm{N}}\right)}^{1/2} $$

6 $$ {\mathrm{R}}_{\mathrm{N}}=\left(\mathrm{fu}/\mathrm{B}:\mathrm{P}\right)\times {CLint}_{u, WL}/\mathrm{Qh} $$

In these equations CLp is the total (bound and unbound) plasma clearance, B:P is the blood to plasma ratio, CLb is the whole blood clearance, Qh is the hepatic blood flow, ER is the hepatic extraction ratio, F_H_ is the hepatic availability, fu is the unbound drug fraction in plasma, CLint_,u,WL_ is the whole liver unbound intrinsic clearance, R_N_ is the efficiency number and D_N_ is the dispersion number, which was taken to be 0.17 (23).

In the dispersion model, CLp values can be directly derived from PK data of intravenously administered drugs. In the paediatric population, the unbound drug fraction in plasma (fu) and the blood to plasma ratio (B:P) of a drug can be obtained experimentally and, assuming these values were obtained precisely and accurately, they were fixed in the model (equation 1–6). Qh and CLint_,u,WL_ are thereafter the only two parameters that remain to be estimated. As in this case the dispersion model can be written as a single equation with two unknowns, clinical data on the PK of one drug will not yield structural identifiability. Therefore clinical trial scenarios were explored based on obtaining PK profiles of two different drugs, in which case there would be two equations for clearance, each with two unknowns. Global structural identifiability can then be obtained if the unknowns are the same in both equations.

Using this approach, Qh is a system-specific parameter that will be the same for two drugs administered to individuals from the same population, assuming the drugs do not alter Qh. CLint_,u,WL_ is however a parameter that combines system-specific properties (i.e. isoenzyme abundance) and drug-specific properties (i.e. isoenzyme activity measured in *in vitro* systems as intrinsic clearance (μl / min) per functional unit of system). This means that even when two drugs are metabolized by the same isoenzyme, their CLint_,u,WL_ value is likely to be different. Therefore a re-parameterization of CLint_,u,WL_ was performed to separate system-specific parameters from drug-specific parameters.

The ratio in CLint_u,WL_ (CLint_ratio_) of drug A and drug B that are metabolized by the same isoenzyme is equivalent to the ratio in the activity for the metabolizing isoenzyme of the two drugs according to equation 7. Therefore, this parameter is a drug-specific parameter.

7 $$ {\mathrm{CLint}}_{\mathrm{ratio}}=\frac{{\mathrm{CLint}}_{\mathrm{u},\mathrm{WL}\_\mathrm{B}}}{{\mathrm{CLint}}_{\mathrm{u},\mathrm{WL}\_\mathrm{A}}}=\frac{\mathrm{isoenzyme}\ \mathrm{activity}\ \mathrm{B}\times \mathrm{isoenzyme}\ \mathrm{abundance}\ }{\mathrm{isoenzyme}\ \mathrm{activity}\ \mathrm{A}\times \mathrm{isoenzyme}\ \mathrm{abundance}} = \frac{\mathrm{isoenzyme}\ \mathrm{activity}\ \mathrm{B}}{\mathrm{isoenzyme}\ \mathrm{activity}\ \mathrm{A}} $$

This ratio does not vary with age, as the isoenzyme activity towards a drug is believed to be unaffected by maturational processes. When fixing CLint_ratio_ obtained for the two drugs, one unique CLint_u,WL_ value can be estimated in a patient population for the two drugs according to the re-parametrization in equations 8 and 9:

8 $$ {\mathrm{CLint}}_{\mathrm{u},\mathrm{WL}}={\mathrm{CLint}}_{\mathrm{u},\mathrm{WL}\_\mathrm{A}} $$

9 $$ {\mathrm{CLint}}_{\mathrm{u},\mathrm{WL}\_\mathrm{B}}={\mathrm{CLint}}_{\mathrm{u},\mathrm{WL}}\times {\mathrm{CLint}}_{\mathrm{ratio}} $$

### Step 2: Optimal Design for Precise Population Parameter Estimates

Because population PK modelling disentangles inter-individual variability in PK parameters from residual unexplained variability, this method allows for the estimation of population PK parameter values Θ and their inter-individual variability ω^2^ which is characterized as described in equation 10. In order to obtain precise estimates of Θ and ω^2^ for CLint_,u,WL_ and Qh_,_ a global sensitivity analysis of the uncertainty of these parameters with respect to the drugs ERs was undertaken using PFIM 4.0 (see under uncertainty section in methods). Defining the relationship between parameter uncertainty and the drugs ERs enables optimization of the trial design, by selecting drugs based on their ER. ER was chosen as the trial design parameter to optimize because it reflects the relative information content of drug clearance values regarding CLint_u,WL_ and Qh (See Fig. 1).

#### Clinical Trial Design

**Fig. 2:**
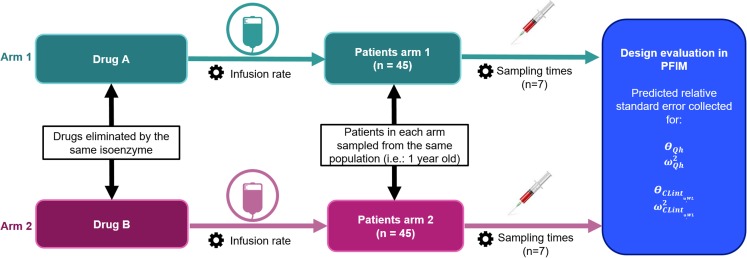
Overview of the clinical trial design. Qh is the hepatic blood flow, CLint_,u,WL_ is the whole liver unbound intrinsic clearance as define in equations 8 and 9. Θ represents the fixed effect and ω^2^ represents the inter individual variance. Cogs indicate trial design variables which are adapted to the drug properties in order to prevent them from being confounding factors when assessing the impact of the drug’s ER on the performance of the design.

The clinical trial design is illustrated in Fig. 2. To allow for structural identifiability, the clinical trial design was composed of two arms. The patients in each arm belonged to the same paediatric population, for which 1-year-olds were chosen, and the drugs administered in each arm were eliminated by the same isoenzyme and had different extraction ratios. In order to ensure that the sampling design was informative enough, while only focusing on the optimization of drug’s ER, the number of patients and blood samples were chosen to be high, with 45 patients included in each arm and 7 blood samples drawn in each patient. This choice allowed us to assess the impact of the drug’s ER on the performance of the trial design without a confounding impact of patient or sample number. Finally, in order to assess the impact of the drug’s ER on the performance of the design without any confounding impact of sampling times, drug dosing, and number of patients and sampling times, these parameters were adapted to the drugs’ properties. To do so, drugs were administered as a constant rate infusion with infusion rate and sampling times adapted to the drugs clearance and half-life respectively, and each arm of the study included the same number of patients and sampling times. The infusion rate was set to reach the same arbitrary steady state concentration of 70 mg/L. Sampling times were drawn every half-life, from the first half life after the start of the infusion up to seven half-lives.

#### Hypothetical Drug Combinations

A total of 99 hypothetical drugs were generated and each unique combination was tested in the design. In order to reduce computational costs and increase interpretability of the results, fu and B:P were taken to be 1. Using the dispersion model, population values of CLint_u,WL_(${\Theta}_{{\mathrm{CLint}}_{\mathrm{u},\mathrm{WL}}}$) were calculated to be such that ER values ranged from 0.01 to 0.99. This required population values of Qh (Θ_Qh_) which was obtained by simulating 500 male and 500 female individuals of 1 year in Simcyp and estimating the population parameter value using the “fitdist” function from the fitdistrplus R package (24) in R.

#### Studied Models and Parameters

For illustration, the proposed workflow is applied to identifying Qh and CLint_,u,WL_ in the paediatric population. As none of the parameters in the dispersion model are impacted by absorption or distribution processes, it is possible to study this PBPK sub-model in full while simplifying the remaining aspects of the PK model for the purpose of this work. Therefore, for each drug in the design (drug A and B), the structural model was a one compartment PK model with a volume of distribution VA and VB respectively and with constant intravenous infusion (see PFIM model code in supplementary material 2 and corresponding model equations in supplementary material 1). Clearance was parameterized as defined by the dispersion model (equation 1–6). CLint_u,WL_ for drug A and B was parameterized according to equations 8 and 9 respectively. The differential equations of the models, as implemented in PFIM can be found in supplementary material 2.

Inter-individual variability was implemented on all fixed effects except on fu, B:P, D_N_ and CLint_ratio_. Inter-individual variability on fu and B:P can be measured and are therefore attainable parameters. D_N_ is a model specific parameter with a fixed value taken from literature. And as a drug-specific parameter, CLint_ratio_ is not subject to inter-individual variability. For the other parameters, inter-individual variability was assumed to follow a log normal distribution and implemented using equation 10, in which η is normally distributed with a mean of 0 and a variance of ω^2^ (see eq.10)

10 $$ Pi=\uptheta \times {e}^{\upeta} $$

A proportional residual error model for each arm in the design with a standard deviation σ of 0.1 was assumed, to yield the same impact of the model error on the parameter uncertainty between study arms and study designs.

Information on the values and estimation of the fixed effects, inter-individual variability and residual errors implemented in the models can be found in Table I.

**Table I:** Estimated and Fixed Model Parameters

Parameters	Values	Estimated and fixed parameters
Fixed effects		
${\Theta}_{{\mathrm{CLint}}_{\mathrm{u},\mathrm{WL}}}$	[0.10–156] L/h	Estimated
Θ_*Qh*_	20.3 L/h	Estimated
Θ_*fu*_	1	Fixed
Θ_*B* : *P*_	1	Fixed
${\Theta}_{{\mathrm{V}}_{\mathrm{A}}}$	4 L	Estimated
${\Theta}_{{\mathrm{V}}_{\mathrm{B}}}$	4 L	Estimated
${\Theta}_{{\mathrm{D}}_N}$	0.17	Fixed
${\Theta}_{{\mathrm{CLint}}_{\mathrm{ratio}}}$.	[0.00065–0.97000]	Fixed
Inter-individual variability		
${\upomega}_{{\mathrm{CLint}}_{\mathrm{u},\mathrm{WL}}}^2$	[0.1–0.8]	Estimated
${\upomega}_{Qh}^2$	[0.1–0.6]	Estimated
${\upomega}_{{\mathrm{V}}_{\mathrm{A}}}^2$	0.25	Estimated
${\upomega}_{{\mathrm{V}}_{\mathrm{B}}}^2$	0.25	Estimated
Residual error		
**σ**_*A*_	0.1	Estimated
**σ**_*B*_	0.1	Estimated

Θ fixed effect; ω^2^ inter-individual variance. ${\Theta}_{{\mathrm{V}}_{\mathrm{A}}}$, ${\Theta}_{{\mathrm{V}}_{\mathrm{B}}}$, correspond to fixed effects for the volume of distribution of drug A and B respectively

#### Design Evaluation with PFIM 4.0

The models, parameters and designs described above were implemented in PFIM 4.0 (19). Concentration-time profiles obtained for both drug A and B were simultaneously analysed to enable for the estimation of Θ and ω^2^ for CLint_,u,WL_ and Qh. The clinical design parameter to optimize for precise estimation of these parameters was the ER of each of the two drugs in the design. To do so, the population Fisher information matrix (FIM) was evaluated for each drug combination in PFIM 4.0. The expected standard errors for each population parameter were calculated as the square root of the diagonal of the inverse of the FIM and reported in PFIM outputs with corresponding relative standard errors (rse). Parameter uncertainties were assessed using their rse with precise estimates being defined as having an rse ≤ 30%.

#### Uncertainty

ER, which is the design variable that was optimized, is dependent on the relative contribution of CLint_,u,WL_ and Qh to overall hepatic metabolic clearance, but not on their absolute values since there is an infinity of combinations of CLint_,u,WL_ and Qh values leading to a specific value of ER. Hence, combinations of ${\Theta}_{{\mathrm{CLint}}_{\mathrm{u},\mathrm{WL}}}$ and Θ_Qh_ that lead to similar ER values are estimated with the same rse%. Therefore no uncertainty on the ${\Theta}_{{\mathrm{CLint}}_{\mathrm{u},\mathrm{WL}}}$ and Θ_Qh_ was implemented in the design evaluation procedure to avoid unnecessary computations, meaning that only one value for ${\Theta}_{{\mathrm{CLint}}_{\mathrm{u},\mathrm{WL}}}$ and one value for Θ_Qh_ as displayed in Table I was used in the PFIM runs.

For each combination of hypothetical drugs, the analysis was repeated for different values of ${\upomega}_{{\mathrm{CLint}}_{\mathrm{u},\mathrm{WL}}}^2$and ${\upomega}_{\mathrm{Qh}}^2$ in order to account for uncertainty in these parameters. The range in ${\upomega}_{{\mathrm{CLint}}_{\mathrm{u},\mathrm{WL}}}^2$ was set from 0.1 to 0.8, while the range in ${\upomega}_{\mathrm{Qh}}^2$ was set from 0.1 to 0.6, both reflecting realistic ranges (see supplementary material 1). Then, design evaluations were repeated using each combination of the upper and lower uncertainty value of the defined range for ${\upomega}_{{\mathrm{CLint}}_{\mathrm{u},\mathrm{WL}}}^2$ and ${\upomega}_{\mathrm{Qh}}^2$, running 4 possible inter-individual variance scenarios in total yielding a global sensitivity analysis of the uncertainty of CLint_,u,WL_ and Qh. Only the lowest and highest values for ${\upomega}_{{\mathrm{CLint}}_{\mathrm{u},\mathrm{WL}}}^2$and ${\upomega}_{\mathrm{Qh}}^2$ were retained in the uncertainty analysis since this yields the extreme values for the rse values. Rse results of all variance scenarios were collapsed for each hypothetical drug combination into one single precision category reflecting the worst case scenario of all variance scenarios. The PFIM model file, input file, and the R command to launch the PFIM evaluation runs for all combinations of hypothetical drugs for one uncertainty scenario are provided in supplementary material 2 to 4. The table containing the drug properties used for the PFIM runs are provided in supplementary table 1.

### Step 3: Investigation of Performance of the Proposed Design

In step 2, the requirements for ER of the two drugs yielding numerical identifiability of fixed effect Θ and inter individual variability ω^2^ of CLint_,u,WL_ and Qh were identified. In the last step of the developed approach, the performance of the proposed design was investigated in terms of bias and imprecision using stochastic simulation and estimation (sse).

First, two drugs meeting the ER requirements defined in step 2 were selected and their ER was converted to expected plasma clearance (CLp) values in a one year old using the expected age-appropriate Θ_Qh_ value derived from Simcyp in step 2. The volume of distribution of each drug was used together with their expected CLp to derive their expected half-life. Since the clinical trial design depends on the half-life and CLp of the two drugs for establishing sampling times and infusion rate respectively these parameters were used for its implementation.

Because the true value of Θ_Qh_ might differ from the value derived from Simcyp in step 2, the selected drugs might have an ER differing from their expected ER. Therefore, uncertainty in both Θ and ω^2^ values of CLint_,u,WL_ and Qh, were accounted for in the sse. This was performed by sampling each of these parameters 1000 times from a uniform distribution. For the population parameters the range of the distribution entailed the typical values from step 2 ± 50%. For the variance parameters the range of the distribution entailed the range defined for the parameter uncertainty in step 2. Sampling of population parameter values Θ_*Qh*_ and ${\Theta}_{{\mathrm{CLint}}_{\mathrm{u},\mathrm{WL}}}$ was restricted, such that their corresponding CLp values yielded a variation of ± 30% of the drugs’ CLps value derived from the ERs of the selected drug A and drug B. This mimics the accepted 30% uncertainty of the reported CLp value in literature. The performance of the design settings were assessed using relative estimation error re (equation 11), mean relative estimation error mre (equation 12), and and relative root mean square error rrmse (equation 13) for Θ and ω^2^ CLint_,u,WL_ and Qh, using only the runs for which minimization was successful.

11 $$ \mathrm{Relative}\ {\mathrm{estimation}\ \mathrm{error}}_i\ \left(\%\right)=\frac{est_i-{true}_i}{true_i}\times 100 $$

12 $$ \mathrm{Mean}\ \mathrm{relative}\ \mathrm{estimation}\ \mathrm{error}\ \left(\%\right)=\frac{1}{N}{\sum}_{i=1}^N\frac{es{t}_i- tru{e}_i}{tru{e}_i}\times 100 $$

13 $$ \mathrm{Relative}\ \mathrm{root}\ \mathrm{mean}\ \mathrm{square}\ \mathrm{error}\ \left(\%\right)=\sqrt{\frac{1}{N}{\sum}_{i=1}^N\frac{{\left( es{t}_i- tru{e}_i\right)}^2}{tru{e_i}^2}\times 100} $$

In these equations, *true* is the true parameter value used in the simulation step, *est* is the estimated parameter value from the estimation step, and *i* is the run index, ranging from 1 to N, the total number of runs for which minimization was successful.

## Results

### Step 1: Structural Identifiability

Qh and CLint were found to be structurally identifiable when the concentration-time profiles from a clinical trial with 2 arms were simultaneously analysed. Each arm of the trial required patients from the same population and administration of different drugs metabolized by the same isoenzyme for which the ratio in intrinsic clearance (CLint_ratio_) is known.

### Step 2: Optimal Design for Precise Population Parameter and Variance Estimates

**Fig. 3:**
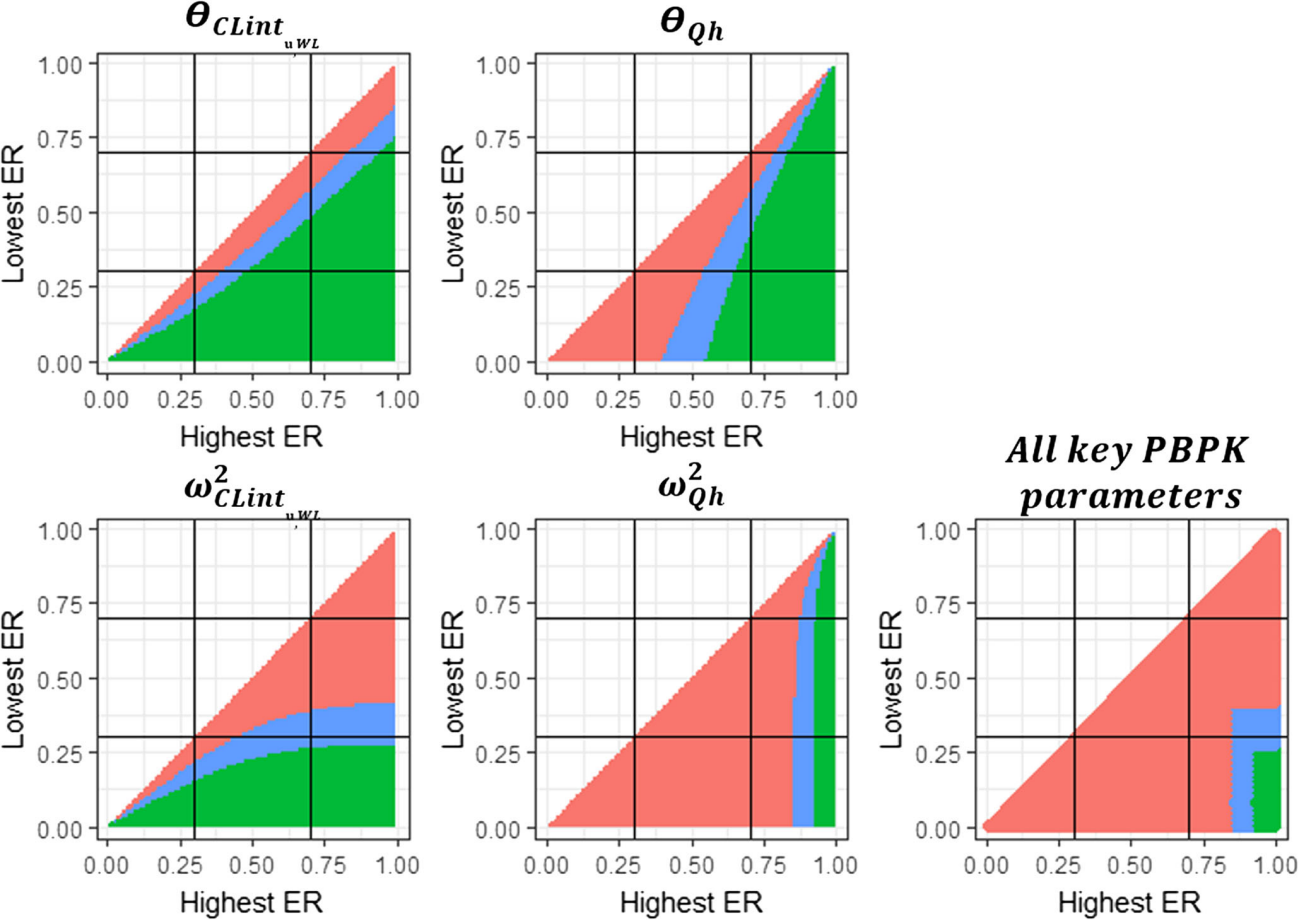
Parameter precision as a function of the extraction ratios (ER) of the two drugs studied in the clinical trial. Θ represents the fixed effect, ω^2^ the inter-individual variance in the parameter. CLint_,u,WL_ is the whole liver unbound intrinsic clearance as defined in equations 8 and 9, Qh is the hepatic blood flow. For each tested drug combination, the precision of the parameter estimates are summarized, with each pixel representing the results for all four variance scenarios (i.e.: ${\upomega}_{{\mathrm{CLint}}_{\mathrm{u},\mathrm{WL}}}^2$ and ${\upomega}_{\mathrm{Qh}}^2$ of 0.1 and 0.1, 0.1 and 0.6, 0.8 and 0.1 or 0.8 and 0.6 respectively) according to the following colour scheme: green indicates relative standard errors (rse%) ≤ 30% for all scenarios, blue indicates rse% is between 30 and 50% for at least one variance scenario, and red indicates rse% > 50% for at least one variance scenario.

Precision of fixed effects and variance values for CLint_,u,WL_ and Qh obtained with combinations of two drugs in the described clinical trial design are summarized in Fig. 3. Except for drug combinations with similar extraction ratio ER (i.e. drug combinations near the line of unity), most drug combinations will lead to a precise ${\Theta}_{{\mathrm{CLint}}_{\mathrm{u},\mathrm{WL}}}$estimate. However, for the estimate of ${\upomega}_{{\mathrm{CLint}}_{\mathrm{u},\mathrm{WL}}}^2$ to be precise, at least one of the drugs should have a low extraction ratio. For Θ_Qh_, most drug combinations leading to precise estimation include one drug with a high ER, while for precise estimation of ${\upomega}_{\mathrm{Qh}}^2$, at least one drug with an ER ≥ 0.93 is required. Finally, precise estimation of all parameter estimates (rse ≤30%) requires one drug with an ER ≥ 0.93 and one drug with an ER ≤ 0.27. Parameter estimation with rse ≤ 50% (corresponding to the blue and green areas in Fig. 3) for all parameters, requires one drug with an ER ≥ 0.85 and one drug with an ER ≤ 0.41.

Supplemental fig. 1 shows the results presented in Fig. 3, but separated for the four variance scenarios. As shown by both Fig. 3 and supplementary figure 1, ${\upomega}_{{\mathrm{CLint}}_{\mathrm{u},\mathrm{WL}}}^2$and ${\upomega}_{\mathrm{Qh}}^2$ drive the threshold for the highest and lowest ER value required in the drug combination for precise estimation of all parameters respectively. Supplementary figure 1 also shows that the value of each of these thresholds are driven by both ${\upomega}_{{\mathrm{CLint}}_{\mathrm{u},\mathrm{WL}}}^2$and ${\upomega}_{\mathrm{Qh}}^2$. For precise parameter estimates, an increase in ${\upomega}_{\mathrm{Qh}}^2$ from 0.1 to 0.6 leads on one hand to a decrease in the threshold for the highest ER value required in the drug combination from 0.79 to 0.56 when ${\upomega}_{{\mathrm{CLint}}_{\mathrm{u},\mathrm{WL}}}^2$ is equal to 0.1 and from 0.93 to 0.80 when ${\upomega}_{{\mathrm{CLint}}_{\mathrm{u},\mathrm{WL}}}^2$is equal to 0.8. On the other hand, such an increase in ${\upomega}_{\mathrm{Qh}}^2$ also leads to a decrease in the threshold for the lowest ER value required in the drug combination from 0.48 to 0.27 when ${\upomega}_{{\mathrm{CLint}}_{\mathrm{u},\mathrm{WL}}}^2$ is equal to 0.1 and from 0.76 to 0.55 when ${\upomega}_{{\mathrm{CLint}}_{\mathrm{u},\mathrm{WL}}}^2$is equal to 0.8. The reverse is observed regarding ${\upomega}_{{\mathrm{CLint}}_{\mathrm{u},\mathrm{WL}}}^2$, with an increase in ${\upomega}_{{\mathrm{CLint}}_{\mathrm{u},\mathrm{WL}}}^2$leading to an increasing threshold for the highest ER value required in the drug combination for precise estimates of ${\upomega}_{\mathrm{Qh}}^2$ and Θ_Qh_, and an increasing threshold for the lowest ER value required in the drug combination for precise ${\upomega}_{{\mathrm{CLint}}_{\mathrm{u},\mathrm{WL}}}^2$ estimates.

The results on ER requirements for drug A and B are independent of the age of children as the ER does not depend on absolute values of Θ_Qh_ and ${\Theta}_{{\mathrm{CLint}}_{\mathrm{u},\mathrm{WL}}}$. However, the ERs of drugs are often unknown, especially in special patient populations, because as presented in Fig. 1, ER in children changes due to maturation of both CLint_,u,WL_ and Qh and the impact of these changes are isoenzyme-dependent due to different maturation patterns.

To facilitate the identification of model drugs for which clinical data could be obtained to precisely estimate population values and variance of CLint_,u,WL_ and Qh at different ages, maturation patterns of different isoenzymes were used to identify isoenzymes and ages for which drugs with the lowest and highest ER required for such estimation are likely to exist. To do so, the dispersion model in combination with maturation patterns for CLint_,u,WL_ of various isoenzymes as implemented in Simcyp V15 (see supplementary material 1) were used to calculate expected ER values for the hypothetical drugs that are substrates of these isoenzymes in children of various ages. We found that drugs with an ER ≤ 0.27 are likely to exist for all investigated isoenzymes at all ages (results not shown). However, drugs with an ER ≥ 0.93 are likely to exist only for specific isoenzyme pathways and ages, as shown in Table II by the increased blue and red boxes with younger ages for most isoenzyme pathways. This is, because low enzyme maturation will reduce the overall ER of drugs. As a result, drugs metabolized by slowly maturing isoenzymes such as CYP2E1 and UGT2B7, are unlikely to have a high ER at young ages. On the other hand, drugs metabolized by fast maturing isoenzymes, such as CYP1A2 or UGT1A4, with a very high ER over a wide range of paediatric ages are likely to exist and could be used as model drugs.

**Table II:**
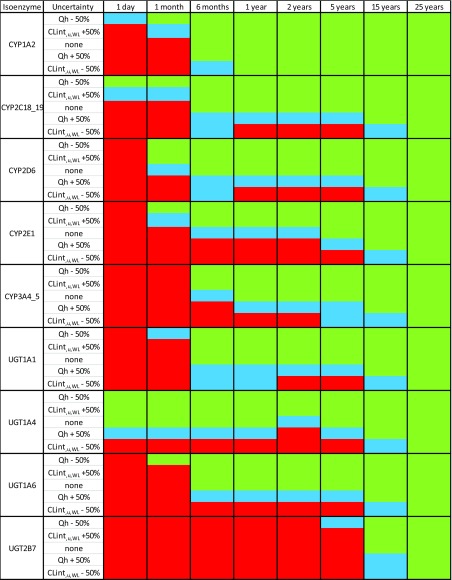
Identification of Isoenzyme Pathways and Ages for which Combinations of Two Drugs Leading to Precise Qh and CLint_,u,WL_ Fixed Effects and Inter-Individual Variability Estimates is Theoretically Possible

The cells indicate whether within the set of hypothetical drugs (see supplementary material 1) a combination of two drugs exist with which Qh and CLint_,u,WL_ fixed effects and inter-individual variability estimates can be obtained with rse ≤30% (green), 30% < rse ≤50% (blue), or rse > 50% (red). Calculations are performed for a situation in which Θ_Qh_ and isoenzyme maturation are set to values as implemented in Simcyp V15 or to a value that is 50% higher or lower reflecting extreme scenarios of uncertainty on Θ_Qh_ and ${\Theta}_{{\mathrm{CLint}}_{\mathrm{u},\mathrm{WL}}}$.

For convenience, to identify drugs with desired ER values, the ER values have also been translated into the required total plasma clearance values (CLp) for various paediatric ages. This is presented in Table III, which shows for instance that in a one year old child, ER ≤ 0.27 and ER ≥ 0.93 translate into a CLp ≤ 5.5 L/h and a CLp ≥ 18.9, respectively.

**Table III:** Conversion Table to Convert the Extraction ratio (ER) Values of 0.27 and 0.93 to Total Plasma Clearance (CLp, equation 1) in Various Postnatal Ages Using Expected Hepatic Blood Flow (Qh) Values and fu and B:*P* Values Taken to be 1

	Expected Θ_Qh_ (L/h)	CLp (L/h) corresponding to ER = 0.27	CLp (L/h) corresponding to ER = 0.93
25 years	87.0	23.5	80.9
15 years	89.2	24.1	83.0
5 years	41.5	11.2	38.6
2 years	30.0	8.1	27.9
1 year	20.3	5.5	18.9
6 months	13.0	3.5	12.1
1 month	7.5	2.0	7.0
1 day	6.4	1.7	6.0

Expected Θ_Qh_ is the fixed effect for the hepatic blood flow derived from Simcyp simulations

### Step 3: Investigation of Bias

The performance of a clinical trial design resulting from step 1 was evaluated using sse with one drug with an ER of 0.94 and one drug with an ER of 0.2 in one year old children. The uncertainty on ${\Theta}_{{\mathrm{CLint}}_{\mathrm{u},\mathrm{WL}}}$ and Θ_*Qh*_ included in the sse, lead to the simulation of 1000 combinations of ${\Theta}_{{\mathrm{CLint}}_{\mathrm{u},\mathrm{WL}}}$ and Θ_*Qh*_with a range of ER from 0.09 to 0.38 and from 0.76 to 0.99 for the drug with the lowest and the highest ER respectively. Minimization was successful in 78.3% of the sse runs. The mre was ≤7.5% for all parameters (Table IV), and the rrmse was below 31%. The confidence interval (5th and 95th percentile) of the re was below or around 20% for ${\Theta}_{{\mathrm{CLint}}_{\mathrm{u},\mathrm{WL}}}$ and Θ_Qh_. Higher values were found for ${\upomega}_{{\mathrm{CLint}}_{\mathrm{u},\mathrm{WL}}}^2$and ${\upomega}_{\mathrm{Qh}}^2$, with values below or around 47%.

**Table IV:** Assessment of the Clinical trial Performance Based on the Precision and Accuracy of the Parameter Estimates of the sse with Uncertainty

	${\Theta}_{{\mathrm{CLint}}_{\mathrm{u},\mathrm{WL}}}$	Θ_*Qh*_	${\upomega}_{{\mathrm{CLint}}_{\mathrm{u},\mathrm{WL}}}^2$	${\upomega}_{Qh}^2$
re 5th percentile	−16.2935	−18.356	−44.4268	−47.0063
re 50th percentile	0.715404	−1.6823	−9.7909	−10.7792
re 95th percentile	23.68881	16.63842	41.03665	46.80308
mre	1.548568	−1.19669	−7.38877	−7.24315
rrmse	12.21483	10.9555	26.24813	30.54991

Θ fixed effect, ω^2^ inter-individual variance. CLint_,u,WL_ is the whole liver unbound intrinsic clearance as define in equations 8 and 9 and Qh is the hepatic blood flow

*RE* relative estimation error; *MRE* mean relative estimation error; *RRMSE* relative root mean square error

## Discussion

The aim of this paper was to develop an analysis framework to investigate the feasibility and clinical trial requirements for the estimation on clinical data of PBPK parameters with a population PK approach. This work represents, to our knowledge, the first application of population optimal design principles for the estimation of PBPK parameters. Being able to *a priori* define trial requirements that yield sufficiently informative data (i.e. numerical identifiability), is essential for a decision making process when costs and benefits of performing a study need to be weighed. The complex design requirements derived in our example for instance, would not be easy to define and would likely not have been met using conventional study design approaches. The execution of clinical trials that yield uninformative data that do yield numerical identifiability is both unethical, especially in vulnerable populations, and cost-inefficient.

While we focused on CLint_,u,WL_ and Qh in a paediatric population, the analysis workflow herein developed can be applied to other PBPK parameters, a different number of parameters, and for other populations. In these cases, the workflow would contain the same steps as outlined here: structural identifiability, optimal design and evaluation of the optimized design performance.

In our example, in the first step of the workflow, structural identifiability for the estimation of both CLint_,u,WL_ and Qh was found possible when PK data of a minimum of two drugs were simultaneously analysed. In addition, both drugs must be administered in patients groups from the same population, metabolized by the same isoenzyme, and their CLint_ratio_ should be known. CLint_ratio_ is the ratio of the *in vitro* measured intrinsic clearance of the drugs and is a drug-specific parameter (equation 7).

In the second step, the use of optimal design not only allowed to optimize the characteristics of drugs to include in the clinical trial design in order to solve numerical identifiability issues, but also acts as a safeguard ensuring global identifiability of the model parameters. Indeed, optimal design identifies models that are either globally or locally structurally non identifiable since their Fisher information matrix is non invertible (25). We found that given the described trial design, the two drugs included in the trial should have an ER ≥ 0.93 and an ER ≤ 0.27, in order to precisely (rse ≤ 30%) estimate Θ and ω^2^ for CLint_,u,WL_ and Qh. These requirements might not be easy to meet since drugs with ER ≥ 0.93 are rare, especially in very young children whose isoenzyme maturation can lead to a decrease in ER with decreasing age (26). This is shown in Table II, displaying isoenzymes and ages for which drugs with ER values that meet these criteria exist within the set of hypothetical drugs tested. These results show that characterization of isoenzyme specific ontogeny is challenging in very young children not only due to the sparsity and lack of available data in this population, but also due to intrinsic characteristics of the system which leads to a decrease in ER with decreasing age. Drugs metabolized by fast maturing isoenzymes were found to be the best model drugs to estimate Qh and CLint_,u,WL_ simultaneously. For instance, CYP1A2 substrates are likely to have the required ERs in children as young as 6 month even in situations where ${\Theta}_{{\mathrm{CLint}}_{\mathrm{u},\mathrm{WL}}}$ and Θ_Qh_ deviate up to ± 50% of their expected values_._ UGT1A4 substrates are likely to have the required ERs in children as young as term neonates of one day, but only in situations where ${\Theta}_{{\mathrm{CLint}}_{\mathrm{u},\mathrm{WL}}}$ and Θ_Qh_ values correspond to their expected value or are up to +50% and − 50% of their expected value respectively. To support the selection of such model drugs from existing drugs, the requirements in ER were translated into CLp values in the conversion table (see Table III) using reported hepatic blood flow values. Overall, these results do highlight the importance of investigating the clinical trial requirements *a priori*, as otherwise the chances of successfully estimating PBPK model parameters from clinical PK data using population approach will be very low.

In the last step of our analysis workflow, stochastic simulations and estimations were used to assess the performance of the optimized study design, since optimal design only addresses parameter precision. While step 2 defined the ER of the two drugs required in the clinical trial, in practice the selected drugs might have an ER deviating from their expected values, which was accounted for in the sse. Uncertainty in fixed effects and inter-individual variability for CLint_,u,WL_ and Qh was also accounted for in the sse. This step allowed for the assessment of whether further investigation to optimize the clinical trial design would be needed to ensure unbiased parameter estimates. Absolute mean ree was below 7.5% for all parameters and the rrmsee was below 31%, meaning that on average the estimates both are accurate and precise. The confidence interval (5th and 95th percentile) of the relative estimation error was below or around 20% for ${\Theta}_{{\mathrm{CLint}}_{\mathrm{u},\mathrm{WL}}}$ and Θ_Qh_. Higher values were found for ${\upomega}_{{\mathrm{CLint}}_{\mathrm{u},\mathrm{WL}}}^2$and ${\upomega}_{\mathrm{Qh}}^2$ which was expected due to parameter uncertainty and the general difficulty in estimating inter-individual variability. Overall, the sse showed that the proposed clinical trial design is robust to parameter uncertainty.

Once existing model drugs with the required ER are identified, clinical data to estimate Θ and ω^2^ for CLint_,u,WL_ and Qh can be sought. The source of these data could either be new clinical trials or historical data from studies meeting the defined design requirements.

An advantage of PBPK models is that quantification of system-specific model parameters, like Qh and the maturation profile of CLint_,u,WL_ for each isoenzyme, needs to be performed only once. Once Qh has been quantified for instance using substrates for the CYP1A2 or UGT1A4 isoenzymes, this can be implemented in the PBPK model either as fixed value or as prior in order to estimate Θ and ω^2^ for CLint_,u,WL_ for any other drug or other isoenzyme pathway in the same population. This is important because both CLint_,u,WL_ and Qh cannot be directly measured and because identifiability is important for the estimation of PBPK model parameters (27).

While in this work we focus on popPBPK parameter estimation for a specific age, the scalability of the rse results with regard to ER allows for the applicability of design requirements across the entire age range. Since most paediatric PK studies include patients of a range of ages, estimation of a maturation function would be preferable over the estimation of parameter values for each specific paediatric age. In the future this work could be extended to the design of studies for the estimation of such maturation functions. This will require the *a priori* definition of the most suitable covariate for maturation function and optimization of study designs for the estimation of parameters in continuous covariate functions, the latter of which is not yet available in the PFIM software.

In the developed analytical workflow, optimal design principles were applied in an innovative manner. The clinical design parameter optimized was the ER of the drugs included in the clinical trial. In a classical optimal design setting, optimized clinical design parameters include the number of patients and samples, sampling times, and drug dose. In this work, in order to assess the impact of the drug’s ER on the performance of the design without any confounding impact these design parameters, these parameters were set such as to have the same impact for each hypothetical drug in the study. To do so, drugs were administered as a constant rate infusion with infusion rate and sampling times adapted to the drugs clearance and half-life respectively, and each arm of the study included the same number of patients and number of blood samples. Practically, such design adaptation on half-life and clearance means that when normalizing sampling times over the drug’s half-life, sampling times and measured concentrations are identical between different drugs (between each arm of the design and between different drug combinations tested), which in turn allows for the same impact of sampling times and dosing regimen on the design performance. Another innovative feature of the workflow is that the optimal design is not given as a unique solution as classically performed, but as a range of solutions. This was done by investigating the parameter space of the design variable to optimize (ER) and categorization of the results by level of precision.

The number of patients and samples in the assessed trial designs in step 2 of the workflow were selected to be relatively high, so that these variables would not be limiting to the optimization of trial requirements regarding the definition of ER values. Once the important design features relevant for the research question of interest have been defined in the proposed workflow, traditional optimal design procedures could be applied to further optimize the trial design regarding these variables.

In the developed workflow, computational cost was tremendously reduced through the optimization of a scalable variable and the use of extreme variance scenarios to account for parameter uncertainty. The ER of a drug is a scalable variable, as it can be converted to clearance values in any paediatric age by using the expected hepatic blood flow in the corresponding age which represents the scaling factor. Indeed, ER reflects the relative contributions of Qh and CLint_,u,WL_ to the hepatic metabolic clearance, but is independent of the absolute value of these two parameters. Therefore, the results of the optimal design analysis obtained for one age, one year-olds in our example, can be extended to any other ages, allowing to reduce the computational cost and to facilitate results interpretation. Moreover, the ER summarizes the influence of all drug-specific parameters (e.g.: drug binding to plasma proteins, drug distribution in red blood cells, etc.), and therefore, accounting for uncertainty in ${\Theta}_{{\mathrm{CLint}}_{\mathrm{u},\mathrm{WL}}}$ and Θ_Qh_ becomes unnecessary in the optimal design phase and the result obtained in our example for drugs with an unbound drug fraction in plasma or fu of 1 and blood to plasma ratio or B:P of 1 can be translated to drugs for which these parameters take different values. Testing all combinations of extreme ${\upomega}_{\mathrm{Qh}}^2$ and ${\upomega}_{{\mathrm{CLint}}_{\mathrm{u},\mathrm{WL}}}^2$ values lead to results reflecting the best and worst case scenarios in terms of parameter precision. Because we defined precise parameter estimates as rse < 30% in all tested variance scenarios, the final results reflect the worst case scenarios and account for all untested intermediate variance scenarios.

In conclusion, this work presents an analysis framework that allows for the *a priori* identification of clinical trial requirements that would allow for the estimation of PBPK model parameters from clinical data using population modelling. The example on CLint_,u,WL_ and Qh shows that it may be unlikely to design an adequate clinical trial, without the knowledge obtained by the application of this analysis framework. Being able to identify PBPK parameters that cannot be obtained by direct experimental measurements in a time and cost efficient manner would greatly improve the development of PBPK models and their predictive performance.

## ACKNOWLEDGMENTS AND DISCLOSURES

We would like to thank Sinziana Cristea for reviewing all model codes used in this study. Trevor Johnson is a paid employee of Simcyp Limited. Professor Amin Rostami-Hodjegan holds shares in Certara, a company focusing on Model-Informed Drug Development. This author has completed the Unified Competing Interest form at http://www.icmje.org/coi_disclosure.pdf (available on request from amin.rostami@manchester.ac.uk) and declares no support from any organisation for the submitted work. This study was supported by the Innovational Research Incentives Scheme (Vidi grant, June 2013) of the Netherlands Organization for Scientific Research (NWO) to Catherijne A. J. Knibbe (2013).

## Electronic supplementary material

Supplementary Figure 1 (DOCX 653 kb)

Supplementary material 1 (DOCX 35 kb)

Supplementary material 2 (R 1 kb)

Supplementary material 3 (R 6 kb)

Supplementary material 4 (TXT 963 bytes)

Supplementary table 1 (CSV 4 kb)

## Abbreviations

Θ: Fixed effect
CLint_,u,WL_: Whole liver unbound intrinsic clearance
CLp: Total (bound and unbound) plasma clearance
mre: Mean relative estimation error
PBPK: Physiologically-based pharmacokinetics
Qh: Hepatic blood flow
re: Relative estimation error
rrmse: Relative root mean square error
ω^2^: Inter-individual variance
