# Supplementary material for: Can Population Modelling Principles be Used to Identify Key PBPK Parameters for Paediatric Clearance Predictions? An Innovative Application of Optimal Design Theory
Source: Pharm Res. 2018 Sep 14;35(11):209. doi: 10.1007/s11095-018-2487-1 (PMC6156772; doi:10.1007/s11095-018-2487-1)
Supplement: Supplementary file 1 — (DOCX 653 kb) [file 11095_2018_2487_MOESM1_ESM.docx]

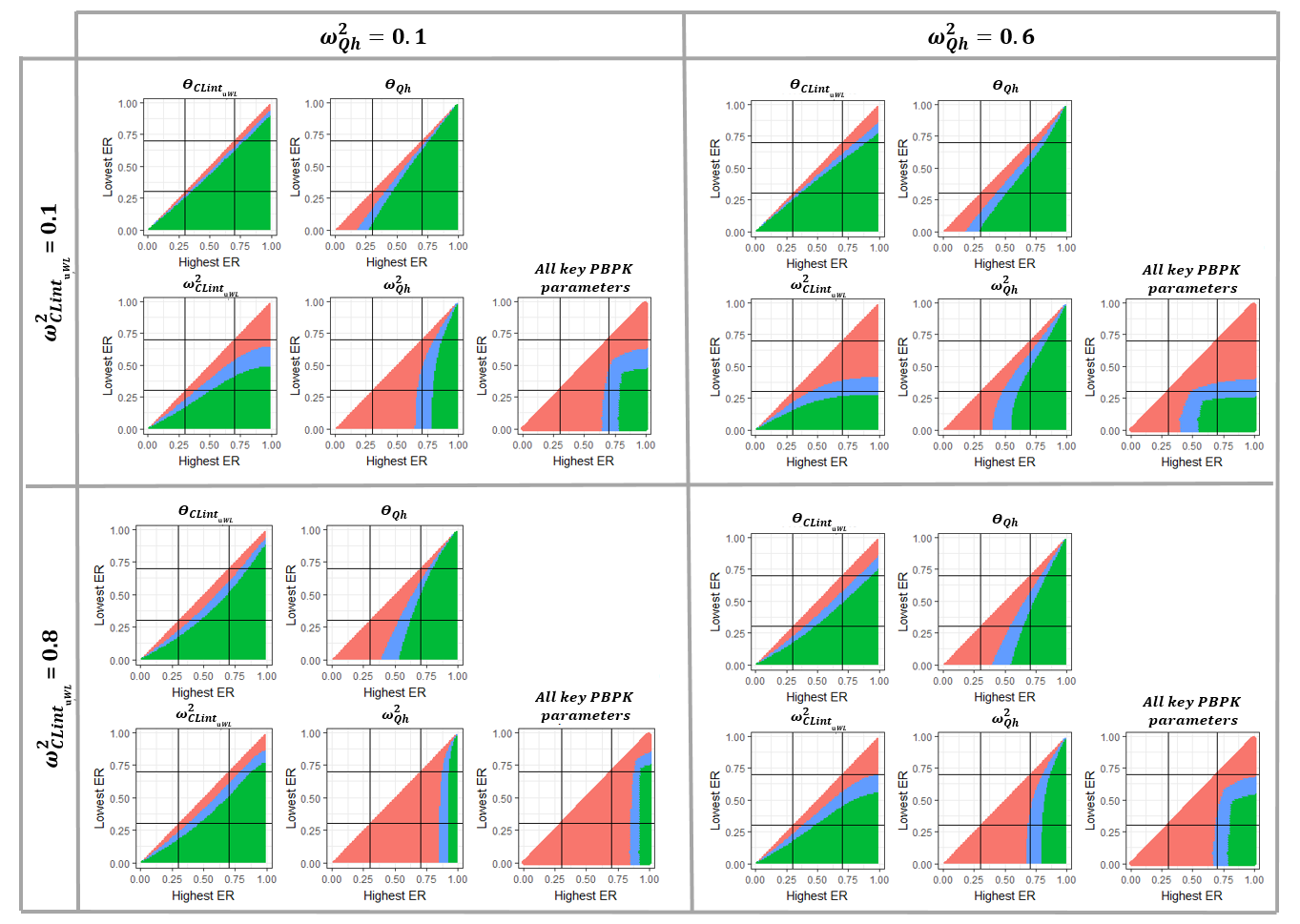
Supplementary Figure 1

**Figure S1** Parameter precision as a function of the extraction ratios (ER) of the two drugs included in the clinical trial design, separated by variance scenario. Each pixel represent a tested drug combination, with the precision of the parameters being summarized as rse%≤30 (green), 30% < rse% ≤ 50% (blue), and rse% > 50% (red).
