# Supplementary material for: Can Population Modelling Principles be Used to Identify Key PBPK Parameters for Paediatric Clearance Predictions? An Innovative Application of Optimal Design Theory
Source: Pharm Res. 2018 Sep 14;35(11):209. doi: 10.1007/s11095-018-2487-1 (PMC6156772; doi:10.1007/s11095-018-2487-1)
Supplement: Supplementary file 2 — 1 (DOCX 35 kb) [file 11095_2018_2487_MOESM2_ESM.docx]

**Supplementary material 1**

**Realistic extraction ratio ranges for various isoenzymes and ages**

To facilitate the identification of existing compounds that would be suitable model drugs, the maximum ranges in extraction ratio that are expected for hypothetical drugs that are substrates for various isoenzymes in children of various ages were calculated. This was done using the dispersion model in combination with maturation patterns of various isoenzymes as implemented in Simcyp V15. ϴ_Qh_ values were used as obtained for each age as displayed in table III and $ϴ_{\mathrm{CLint}_{u,WL}}$ values were computed as described in equation 1.

CLint_,u,WL_ = CLint,mic × MPPGL× liver weight × IO (1)

CLint,mic stands for intrinsic microsomal clearance for which values between 0.56·10^-6^ and 0.209.10^-3^ L.min^-1^.mg^-1^ microsomal protein were used according to literature (1). The population mean of the product of MPPGL (microsomal protein per gram of liver) and liver weight was derived from the Simcyp simulation similarly as for the computation of ϴ_Qh_. Isoenzyme ontogeny (IO) values for the different ages and isoenzymes were taken from Simcyp V15 and are displayed in the table below. IO is expressed as percentage of adult isoenzyme activity.

| Age | 1 day | 1 month | 6 months | 1 year | 2 years | 5 years | 15 years | 25 years |
| --- | --- | --- | --- | --- | --- | --- | --- | --- |
| IO CYP1A2 (%) | 24 | 35 | 118 | 150 | 164 | 161 | 126 | 100 |
| IO CYP2C18-19 (%) | 30 | 33 | 84 | 95 | 97 | 98 | 100 | 100 |
| IO CYP2D6 (%) | 6 | 47 | 84 | 91 | 95 | 98 | 100 | 100 |
| IO CYP2E1 (%) | 10 | 37 | 59 | 67 | 74 | 82 | 88 | 100 |
| IO CYP3A4_5 (%) | 11 | 13 | 48 | 78 | 96 | 104 | 106 | 100 |
| IO UGT1A1 (%) | 0.2 | 23 | 98 | 104 | 100 | 100 | 100 | 100 |
| IO UGT1A4 (%) | 74 | 74 | 74 | 75 | 77 | 81 | 97 | 100 |
| IO UGT1A6 (%) | 15 | 30 | 63 | 76 | 87 | 95 | 100 | 100 |
| IO UGT2B7 (%) | 8 | 9 | 11 | 13 | 18 | 32 | 79 | 100 |

In order to account for uncertainty on Qh and CLint, extraction ratio ranges were also computed with ϴ_Qh_ values of +/50% and with $\theta_{\mathrm{CLint}_{u,WL}}$ values of +/-50% of the values described above. For each isoenzyme, the age range was identified in which drugs with realistic extraction ratio values exist that are required for the precise estimation of Qh and CLint_,u,WL_.

**Definition of realistic ranges for** $\boldsymbol{\omega}_{\mathbf{CLint}_{\mathbf{u,WL}}}^{\boldsymbol{2}}$ **and** $\boldsymbol{\omega}_{\mathbf{Qh}}^{\boldsymbol{2}}$ **for the implementation of parameter uncertainty in PFIM**

In step 2 and 3 of the analytical workflow, uncertainty on $\omega_{\mathrm{CLint}_{u,WL}}^{2}$and $\omega_{\mathrm{Qh}}^{2}$ were implemented using realistic ranges for these parameters.

For $\omega_{\mathrm{CLint}_{u,WL}}^{2}$, the range of variance was set to 0.1-0.8, including the minimum and maximum hepatic $\omega_{\mathrm{CLint}_{u,WL}}^{2}$ estimates derived using Simcyp (0.46 and 0.77 respectively) and reported clearance variance of low extraction ratio drugs (2). For populations of 1 day, 1 month, 6 months, 1, 2, 5, 15 and 25 year-olds, 1000 Simcyp simulations of hepatic CLint_u,WL_ for each population were performed, with 50% females and 50% males, assuming drug metabolism by different isoenzymes. These isoenzymes were isoenzymes for which model drugs have been reported (drugs mainly metabolized by the isoenzyme), namely CYP1A2, CYP2C19, CYP2D6, CYP2E1, CYP3A4-5, UGT1A1, UGT1A4, UGT1A6 and UGT2B7 (3)(4). For each population and isoenzyme, $\omega_{\mathrm{CLint}_{u,WL}}^{2}$ was estimated using the fitdist function from the fitdistrplus R package assuming a log normal distribution.

For Qh a range of variance of 0.1-0.6 was selected, which includes recently reported portal vein flow variance values in healthy adults ($\omega_{\mathrm{Qh}}^{2}$ = 0.13) and in cirrhotic adults (omega = 0.38) using phase contrast MRI sequence with compressed sensing acceleration and high spatial resolution (5). The chosen range also included higher values (0.6 against the highest reported value of 0.38 in adults) in order to account for potentially higher hepatic blood flow variability in paediatric (sub)populations.

**Model equations**

The equations of the pharmacokinetic model (structural model) for drug A and drug B implemented in PFIM in the step 2 of the analytical workflow are given below:

Plasma clearance of drug A

$$RnA=(\mathrm{fuA}/\mathrm{BPA})\times{{CLint}_{u,WL}A}/\mathrm{Qh}$$

$$aA =\sqrt{(1+4RnA\times Dn)}$$

$$CLA =Qh\times BPA\times(1-\frac{4aA}{\left( 1+aA \right)^{2}\exp\left\{ \left( aA-1 \right)/{2D_{N}} \right\}-\left( 1-aA \right)^{2}\exp\left\{ {-\left( aA+1 \right)}/{2D_{N}} \right\}})$$

Plasma clearance of drug B

$$RnB=(\mathrm{fuB}/\mathrm{BPB})\times{{CLint}_{u,WL}A\times{CLint}_{ratio}}/\mathrm{Qh}$$

$$aB =\sqrt{\left( 1+4RnB\times Dn \right)}$$

$$CLB =Qh\times BPB\times(1-\frac{4aB}{\left( 1+aB \right)^{2}\times exp\left\{ \left( aB-1 \right)/{2D_{N}} \right\}-\left( 1-aB \right)^{2}\times exp\left\{ {-\left( aB+1 \right)}/{2D_{N}} \right\}})$$

Plasma concentration time profile of drug A

$\frac{ⅆAA}{dt} = K0A -\frac{CLA}{VA} \times\mathrm{AA}$

$CA(t) = \frac{AA(t)}{VA}$

$\frac{ⅆAB}{dt} = K0B -\frac{CLB}{VB} \times\mathrm{AB}$

$CB(t) = \frac{AB(t)}{VB}$

With estimated parameters VA (volume of distribution for drug A), CLint_u,WL_(whole liver intrinsic unbound clearance of drug A), Qh (hepatic blood flow) and VB (volume of distribution for drug A) and fixed parameters K0A (infusion rate of drug A), K0B (infusion rate of drug B), ${CLint}_{ratio}$ (ratio ${CLint}_{u,WL}B$ /${CLint}_{u,WL}A$), equivalent to the ratio of microsomal intrinsic clearance as could be measured in vitro, fuA (fraction unbound of drug A), fuB (fraction unbound of drug B), BPA (blood to plasma ratio of drug A), BPB (blood to plasma ratio of drug B) and Dn (Dispersion number).

References

1. Nikolic K, Agababa D. Prediction of hepatic microsomal intrinsic clearance and human clearance values for drugs. J Mol Graph Model. 2009 Oct;28(3):245–52.

2. Wang C, Allegaert K, Tibboel D, Danhof M, van der Marel CD, Mathot RAA, et al. Population pharmacokinetics of paracetamol across the human age-range from (pre)term neonates, infants, children to adults. J Clin Pharmacol. 2014 Jun;54(6):619–29.

3. Krekels EHJ, Danhof M, Tibboel D, Knibbe C a J. Ontogeny of hepatic glucuronidation; methods and results. Curr Drug Metab. 2012 Jul;13(6):728–43.

4. Kivistö KT, Kroemer HK. Use of probe drugs as predictors of drug metabolism in humans. J Clin Pharmacol. 1997 Jan;37(1 Suppl):40S–48S.

5. Dyvorne HA, Knight-Greenfield A, Besa C, Cooper N, Garcia-Flores J, Schiano TD, et al. Quantification of Hepatic Blood Flow Using a High-Resolution Phase-Contrast MRI Sequence With Compressed Sensing Acceleration. Am J Roentgenol. 2015 Mar;204(3):510–8.
